# Review of Coping Strategies to Prevent and Mitigate Food Insecurity in Households with Children

**DOI:** 10.1016/j.advnut.2026.100665

**Published:** 2026-05-30

**Authors:** Olivia R Romanovich-Brown, Bethany McGowan, Analí Morales-Juárez, Heather A Eicher-Miller

**Affiliations:** 1 Department of Nutrition Science, Purdue University, West Lafayette, IN, United States; 2 Libraries and School of Information Studies, Purdue University, West Lafayette, IN, United States

**Keywords:** coping strategies, food insecurity, food security, low income, children, adolescents

## Abstract

Food insecurity occurs when household members experience a change in their diets due to limited resources. Compared with all households, food insecurity is more prevalent among those with children in the United States. In approximately half of those households, children did not directly experience food insecurity, indicating that coping strategies, or actions to manage food insecurity, may help prevent this situation by limiting changes to the quality and quantity of the diet. The aim of this scoping review is to summarize and examine documented coping strategies used by low-income households with children in the United States and provide insights for interventions that could improve food security. PubMed, CINAHL, Scopus, and PsycINFO were searched up to January 2026. Two independent reviewers screened articles in Covidence through title/abstract and full-text stages using predefined inclusion and exclusion criteria. Conflicts were reconciled through discussion to reach a consensus. Results and key themes of each study were extracted and synthesized narratively regarding coping strategies to manage food security. Twenty-five studies were identified through the screening process, and 5 key themes were identified: food acquisition, food management, financial coping, reliance on support from family members or friends, and coping strategies implemented by children. Most studies focused on strategies used by mothers and parents, whereas others examined strategies used by the entire household or by children. The findings suggest that multiple members of food-insecure households with children use coping strategies, providing insights for future interventions to improve food security.

The protocol for this review is registered with Open Science Framework, and its registration DOI is 10.17605/OSF.IO/H7TCF.

## Introduction

**Statement of Significance**

Food-insecure households with children implement coping strategies to manage food security. Future nutrition education interventions can promote positive coping strategies to improve the food security of all household members.

Food insecurity is a situation where at least ≥1 people in a household experience changes in their diet or not having enough food because of insufficient resources [1]. Households with children have higher rates of food insecurity compared with all households, and in 2023, 17.9% of households with children in the United States were affected compared with 13.5% of all households [1]. Children may be especially vulnerable to the situation of food insecurity because of the demand for nutrient-dense foods to support optimal growth and development [[2], [3], [4]]. Key markers of poor dietary quality and nutrient inadequacy were linked with food insecurity for both adults and children in previous studies [[5], [6], [7], [8]]. For example, children from food-insecure households had lower intakes of vitamin D, magnesium, and choline compared with those who were food secure, whereas adults in food-insecure households had lower intakes and higher percentages of nutrient inadequacy for magnesium, potassium, and vitamins A, B-6, B-12, C, D, E, and K [7,9].

The classification of food insecurity based on the indications of decreased quantity or quality of food in food-insecure households is also associated with poor child development outcomes, specifically poor physical health indicators like overweight and obesity, reduced bone mass, and iron-deficiency anemia [4,[10], 11], [12]], as well as behavioral and academic indicators such as hyperactivity, aggressive behavior, and frequent school absences [11,[13], [14], [15]]. Adults in food-insecure households similarly have a greater burden of poor mental and physical health compared with their food-secure counterparts because food insecurity is associated with higher rates of stress and depression [16], poor physical health such as anemia, and a higher share of chronic diseases such as cardiovascular disease and obesity [[16], [17], [18], [19]].

Despite common links with poor diet, nutrient intake, and health among children and adults, food insecurity can impact each member of a household in distinct ways because each individual has a different share in the food allocation within the household that may be due to their responsibilities, ages, and relationship to others [[20], [21], [22]]. These diverse positions are also related to various coping strategies, which are actions that each person may try to implement to help maintain food security in the household. For example, parents in food-insecure households may experience stress on their mental and physical health due to feelings of guilt and pressure to provide food for the household [23,24]. To cope with limited food, parents may implement tactics that include rationing, purchasing cheaper foods, relying on extended family members for money, and allocating food to children first [23]. Children, who may have less control over household food and its distribution, may also play a role in coping with food insecurity. For example, children in food-insecure households may take responsibility for managing food resources by participating in parent strategies, generating food resources by seeking support from other family members, and contributing financial resources [22]. Food insecurity can also be experienced differently depending on the age and sex of children within a household. One study found that older and male children reported experiencing very low food security more frequently compared with younger and female children, highlighting potential differences in food allocation and coping strategies, emphasizing age and gender [25].

In about half of the food-insecure households with children in the United States in 2023, children did not directly experience food insecurity, suggesting that coping strategies that caregivers may use to prevent changes to diet in quality or amount may be helpful in limiting the experience of food insecurity [1]. Despite a recognition of common coping strategies, a comprehensive review has not been completed. Aside from the differences in food insecurity prevalence among households with children or adults in the household, it also remains unknown as to whether these strategies are directly linked to maintaining food security. Therefore, this scoping review aimed to summarize and examine the coping strategies used within the household context by both adults and children living in low-income and/or food-insecure situations to attempt to maintain food security and access to healthful foods for both children and adults, and to summarize evidence of the relationship between these coping strategies and food security. The results could lead to the development of new interventions aimed at improving food-security status for all members of a household, potentially having relevance at the individual, household, and national levels.

## Methods

The PRISMA extension for Scoping Reviews (PRISMA-ScR) Checklist was used to inform this scoping review. The search strategy was developed through engaged discussions among the researchers on the project, in consultation with a librarian. We used a standardized keyword-based search strategy, consistent with scoping review guidance emphasizing breadth of literature capture across sources. The search included the following terms: (“Households with children” OR children OR mothers OR fathers OR parents OR guardians OR caregivers) AND (United States) AND (coping OR “coping strategies” OR “family characteristics” OR “mental health”) AND (“food insecurity” OR “food assistance”). The search phrase was applied in the online databases PubMed, CINAHL, Scopus, and PsycINFO in January 2025. To capture any relevant studies published since the initial search, the search phrase was implemented again in January 2026. There were no restrictions on the date of publication, and only studies published in English were eligible. The protocol for this review has been registered with Open Science Framework, and its registration DOI is 10.17605/OSF.IO/H7TCF.

The initial search phrase in January 2025 identified a total of 988 studies across the 4 databases: 483 in PubMed, 267 in Scopus, 121 in CINAHL, and 117 in PsycINFO, which were imported into Covidence, where 319 duplicate references were removed before screening. Two independent reviewers used predefined inclusion and exclusion criteria to evaluate articles identified by the search in both the title/abstract and full-text stages. In the title/abstract phase, reviewers judged each article as “No,” “Maybe,” or “Yes,” where those marked “Maybe” and “Yes” were evaluated in the full-text phase for eligibility. All disagreements between reviewers were resolved through discussion to reach a consensus on the inclusion or exclusion of the article.

**FIGURE 1:**
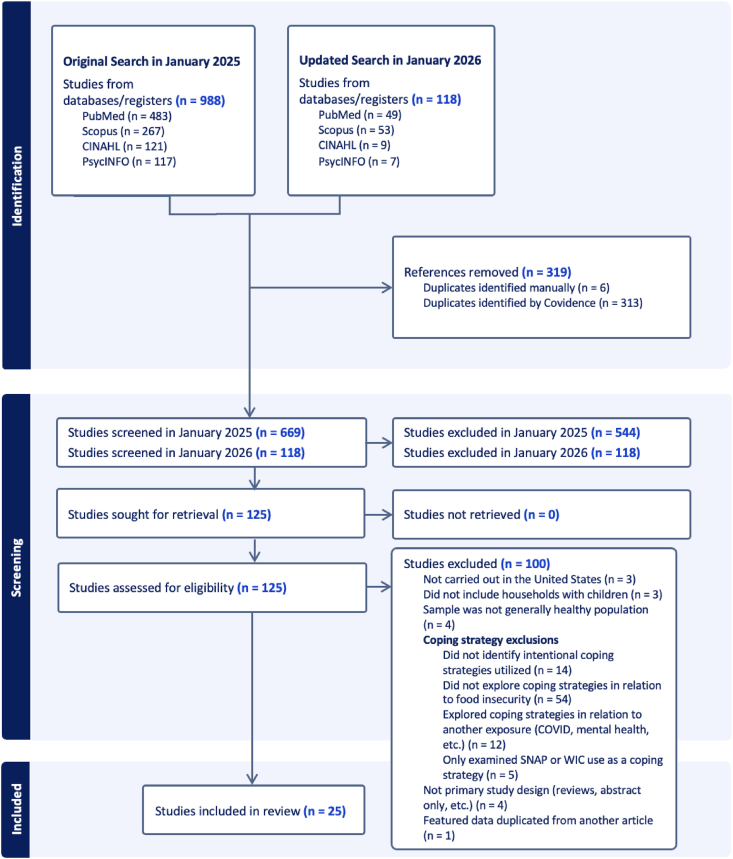
PRISMA Protocols flow diagram of 25 studies exploring coping strategies used by United States households with children to maintain food security. SNAP, Supplemental Nutrition Assistance Program; WIC, Special Supplemental Nutrition Program for Women, Infants, and Children.

Studies were included if they used a primary study design to determine coping strategies used in food-insecure households with children in the United States to maintain food-security status. Studies were excluded if they did not identify intentional coping strategies related to maintaining food-security status or if they focused on techniques in relation to another exposure, or those that exclusively examined the use of federal food assistance programs. There were 669 articles screened in the title/abstract phase, and 125 were assessed for eligibility in the full-text phase. The updated search from January 2026 identified 118 articles screened in the title/abstract phase, with all excluded, and no new studies were added to this review. Twenty-five studies were included in this scoping review, as shown in Figure 1.

For the 25 studies that met the inclusion criteria, data extraction was performed by 2 independent reviewers, and any conflicts were resolved through discussion. Study results were extracted and synthesized narratively into key themes based on the similarity of the action used to maintain household food-security status. The year of publication, title, first author, objective(s), study design, member of household utilizing coping strategies, distinct coping strategies identified, outcomes/findings, and effectiveness of coping strategies were extracted for each study and reported in Table 1 [[22], [23], [24],[26], [27], [28], [29], [30], [31], [32], [33], [34], [35], [36], [37], [38], [39], [40], [41], [42], [43], [44], [45], [46], [47]].

**TABLE 1:** Publication dates, title, first author name, objective, study design, measure of food security or low-income status, population description, member of household using coping strategies, outcomes/findings, and effectiveness of coping strategies for 25 studies exploring coping strategies in food-insecure households with children in the United States to maintain food security.

Title	First author (year of publication)	Objective	Study design; measure of food security or low-income status	Population description	Member of household using coping strategies	Coping strategies identified	Outcomes/findings	Effectiveness of coping strategies
Working to eat: vulnerability, food insecurity, and obesity among migrant and seasonal farmworker families	Kristen Borre (2010) [26]	The purpose of this study was to examine whether or not food insecurity is linked to obesity among adults and children and to broach the question of how the conditions that underlie poor food security contribute to chronic health conditions such as obesity, occupational illness, and injury.	Cross-sectional study, mixed methods; 18-Question United States Household Food-Security Survey Module	The study population was the migrant and seasonal farmworkers who participated in agricultural hired labor during the 2005 growing season in eastern North Carolina and participated in the East Coast Migrant Head Start Program at 2 sites.	Mothers, fathers	Regular work; careful budgeting; participation in programs and services offered; taking food from the field home or eating it unwashed, in the field	Preventing food insecurity required a combination of regular work, careful budgeting, participation in programs and services offered, and dependence on the community for emergency food.	From focus group and individual interviews, periodic hunger and food insecurity were a part of the lifestyle of being a migrant farmworker in the United States, an occupational risk, and the risk was less in the United States than in their home country. Most reported that, although they would like to go home, they could not do so because of a lack of money and regular employment. Opportunities to make money in exchange for hard work were better in the United States, and farmwork fit their cultural lifestyles as migrants and provided the best opportunity they could find, given their abilities and vulnerability. American agriculture needed them, and they needed farmwork to make a living.
Stretching food and being creative: caregiver responses to child food insecurity	Michael P. Burke (2017) [27]	The purpose of this study was to investigate the strategies and behaviors caregivers use to adjust the household food supply in reaction to food insecurity among their children.	Cross-sectional study, qualitative methods; 18-Question United States Household Food-Security Survey Module	Participants were caregivers who reported food insecurity among their children in urban and nonurban areas of South Carolina. Thirty-five percent of the sample had very low food security among children; 61% lived in an urban area; and 80% were African American.	Caregivers	Changes in foods purchased or obtained for household meals; prioritize some foods (e.g., hot dogs, noodles, chicken, rice, beans, potatoes, bread and grits); use foods that can be stretched (e.g., stews, soups, casseroles, pasta, bean or rice dishes); eat more sandwiches to simplify meals; use more canned goods; cut quantity and quality of meats; food banks, church food pantries, food giveaways; borrow food or money (e.g., from family, friends, neighbors); cut some foods (exact foods not specified); more processed and fast foods over fresh foods (e.g., boxed and canned dinners, dollar menus); child food preferences come first; use of federal nutrition assistance programs; garden to supplement food supply; monetary and shopping strategies; buy according to price (e.g., sale, cheapest and store brands); shop at budget grocery stores (e.g., Walmart, Aldi, Save-a-Lot); buy foods in bulk, especially meats; budget finances to stretch funds over a month; use coupons; use utility and other bill money for food purchases; sell plasma for money to buy food; changes to household meal patterns; serve smaller portions at mealtime; reduce adult portions or adult does not eat at all; change meal times and frequency to stretch food; have breakfast foods any time of day; more snacks to ease hunger; first-come, first-served; adaptations in home preparation; be creative, make stuff up or use what we have; make use of leftovers and freeze meals; prepare simpler meals with fewer ingredients; cook less or more; plan meals each week; try to make do, do not cut back	Participants mentioned a total of 1354 strategies and behaviors to adjust the household food supply in reaction to child food insecurity; these were grouped into 5 primary categories (*n =* 735). Changes in foods purchased or obtained for the household’s meals were the most frequent primary strategy or behavior mentioned (*n =* 773) and had the greatest share of total mentions (57%). Monetary and shopping strategies was the second most frequent primary strategy or behavior mentioned (*n =* 245). Changes in household meal patterns was the third most frequent primary strategy or behavior mentioned (*n =* 194; 14%). Adaptations in home preparations was the fourth most frequent primary strategy or behavior (*n =* 130; 9%). Caregivers reported a total of 1365 behaviors related to decreasing or increasing specific foods in their children’s meals; these were grouped into 11 primary categories (*n =* 525).	Not applicable
Teen food insecurity: finding solutions through the voices of teens	Mecca Burris (2020) [28]	The purpose of this study was to identify issues related to food insecurity among teenagers in Tampa Bay, Florida, and aims to better understand the experiences and coping strategies involved with teen food insecurity. The study also generates ideas for improving food assistance programs for teenagers by talking to teens themselves.	Cross-sectional study, qualitative methods; USDA’s Self-Administered Food-Security Survey Module for Children Ages 12 Years and Older	Participants included 38 teens from 5 different communities in Tampa Bay, Florida. Participants were primarily male and ethnically and racially diverse. More than 40% of participant teens were food insecure, with approximately one-third living in households participating in SNAP	Teens	Relying on community; participating in illegal activities; relying on cheap foods; work; relying on teachers or school	Teens employed many creative strategies to deal with or prevent hunger, which sometimes results in socially unacceptable behaviors such as stealing. The most common strategy was to turn to their community. Community included churches, neighbors, friends, and community centers, such as their after-school and summer program sites. Some teens got jobs to provide for themselves or to help their families, and some participate in illegal activities. Stealing food, as well as other necessities such as school supplies, was a common and justified coping mechanism. Selling drugs and “selling themselves” were other illegal activities mentioned, although the teens did not elaborate on what they meant by “selling themselves.” Relying on cheap and convenient food items such as fast food, snack foods, and candy was another way teens dealt with hunger. They explained that these foods were more affordable than a full meal at school or healthy food options (e.g., a McDonald’s hamburger costs less than a salad; skittles only cost $0.75 compared with the $3 school lunch). Lastly, teens relied on help from their teachers. They positively discussed teachers who would provide snacks or lunch food to those who were hungry or did not have money for the school lunch. They explained that some teachers would let them utilize the school food assistance programs more than allowed if needed.	Not applicable
Children are aware of food insecurity and take responsibility for managing food resources	Maryah Stella Fram (2011) [22]	The purpose of this study was to investigate childhood food insecurity and hunger from the child’s own perspective. Because parents were also interviewed (separately), child reports can be considered in the context of household food resources and stressors, parental efforts to manage food insecurity, and other salient aspects of family functioning.	Cross-sectional, qualitative methods; 6-Question Short Form United States Household Food-Security Survey Module	Mothers, children (age 9–16 y), and other household adults in 26 South Carolina families at risk for food insecurity were participants. On the basis of mothers’ responses to the Household Food-Security Module, 16 families experienced low or very low food security in the previous 12 mo	Children, teens	Taking responsibility for managing food resources; participation with adult strategies; initiation of strategies; generation of resources	Study children experienced household food insecurity in 2 components: awareness of food insecurity and taking responsibility for managing food resources. Awareness means that the child had an experience of or an encounter with the household’s food insecurity and understood that experience as being related to not having enough food to meet everyone’s needs. Awareness was further differentiated into 3 subcategories: cognitive, emotional, and physical awareness. The children reported a range of behaviors that reflected taking responsibility for managing household food insecurity. Sometimes this involved participation with adult strategies for stretching resources. Some children went beyond participation to initiate strategies without being asked. Occasionally, children reported that they took responsibility for managing food insecurity by generating more resources themselves.	Not applicable
Questionnaire-based measures are valid for the identification of rural households with hunger and food insecurity	Edward A. Frongillo, Jr. (1997) [29]	The aims of this study were as follows: *1*) to assess the validity of questionnaire-based measures in identifying households with hunger and food insecurity for the purposes of estimating prevalence, targeting, and screening, and *2*) to examine the interrelationships of the questionnaire-based measures of hunger and food insecurity to strengthen our ability to interpret these available measures.	Cross-sectional study, qualitative methods; Radimer/Cornell, Community Childhood Hunger Identification Project, and the NHANES III hunger and food insecurity items	Data were collected in a survey of households containing women with children living at home conducted between January and July of 1993 in the rural county of New York State.	Mothers	Spent low amount on food and no money on eating out; borrowed money for food; applied for food stamps or receive them; free or reduced price school lunch; use of food pantry; could spend less if in a buying club, gardening, hunting/fishing, or obtaining free eggs/milk/meat; spent large amount on food outside the home; >1 adult working; some unemployment but receiving child support, workers’ compensation, or unemployment benefits	Coping strategies were not discussed as an outcome in relation to the study objectives.	Not applicable
Maternal strategies to access food differ by food-security status	Kathleen S. Gorman (2017) [30]	The purpose of this study was to examine factors that may help account for differences between food-secure and food-insecure households within a low-income population. An important question explored in this paper is whether or not household-level behaviors that adults use vary as a function of food-security status. Specifically, the authors examined variability in household food-security status in relation to: *1*) how, when, and where household members shop for food; *2*) other sources of assistance or strategies low-income mothers use when trying to feed their family, and *3*) mothers’ ability to manage household resources.	Cross-sectional study, quantitative methods; 18-Question United States Household Food-Security Survey Module	Participants were 164 low-income mothers of young children (55% Hispanic) from 2 communities in Rhode Island. Over half of the participants were Hispanic, and the majority of participants reported speaking English or both English and Spanish; 21% reported speaking only Spanish. A little over half (56.7%) of households were classified as food secure, and the remaining (43.3%) food insecure; 29.0% had low food security, and 14.1% had very low food security.	Mothers	Using a variety of stores; implementing shopping strategies such as purchasing lower cost food, sales, less junk food, multiple stores, bulk, utilizing shopping list, coupons, and fewer fruits/vegetables; use of nutrition assistance programs, community food programs, food pantry, soup kitchen; informal sources of food, pooling resources, exchanging/trading, store credit, borrowing; visiting restaurants or fast food	In terms of shopping patterns, almost all participants (88%) shopped at least once at a supermarket during the prior week, and 30% shopped at discount stores (Table 3). Far fewer shopped for groceries at corner stores (15%), specialty stores (12%), or convenience stores (10%). Individuals varied widely in how often they reported doing their major food shopping, ranging from daily (2%) to monthly (34%), and the amount of money spent on food varied widely (range 0 to $550 in the past week). When asked about strategies they used (e.g., coupons, buying in bulk, shopping at multiple stores, and using a shopping list), the top 5 most common were purchasing lower cost foods (83%), taking advantage of sales/discount offers (74%), purchasing less junk food (73%), shopping at multiple stores (65%) and buying in bulk (63%). The least common strategy was reducing their purchases of fruits and vegetables (31%). Mothers reported using a mean of 6 (mean = 5.83, SD = 1.94) of the 8 strategies at least some of the time when they shopped. Low-income women in our sample reported utilizing other sources of assistance to increase their ability to provide food for their families. Participants differed in the degree to which they borrowed, pooled their resources, traded, and used credit as alternative food sources.	Not applicable
Food insecurity during pregnancy and breastfeeding by low-income Hispanic mothers	Rachel S. Gross (2019) [31]	The purpose of this study was to conduct semistructured qualitative interviews with low-income Hispanic mothers with children in early infancy, late infancy, and toddlerhood to learn more about their financial pressures and perceived effects on infant and toddler feeding.	Cross-sectional study, qualitative methods; 18-Question United States Household Food-Security Survey Module	Participants were low-income Hispanic mothers (*n =* 100) with infants in the first 2 y of life, all of whom were participants in a randomized controlled trial of an early child obesity prevention intervention. They sampled 100 mothers who were primarily born outside the United States (87%), Spanish-speaking (87%), WIC participants (91%) with infants (32 with 3- to 7-mo-olds, 31 with 10- to 15-mo-olds, and 37 with 19- to 24-mo-olds). The majority reported experiencing food insecurity (67%).	Mothers	Making shopping lists; cooking at home; delaying paying other bills; prioritizing food over other expenses; relying on food staples; relying on social support networks; relying on federal assistance	Home strategies involved considerable planning. Families made shopping lists to prevent unnecessary purchases and food waste (quotation 24), avoided street foods, and cooked at home (quotation 25). Families limited other spending, such as electricity, by turning off televisions and lights (quotation 26) and delayed spending on other bills. Many expressed that “food must be first” and delayed buying clothes and shoes (quotation 27) or paying less important bills, such as the telephone (quotations 28 and 29). Mothers relied on inexpensive staples that stored easily and would not spoil, such as beans, rice, and tortillas (quotations 30–32). Social support networks, such as extended family (quotations 33–35), friends (quotation 36), and churches (quotation 37), provided safety nets when household strategies proved insufficient. Networks either loaned money or directly provided food. Living with extended family allowed for lower rent burden, decreased expenses, and the distribution of bills among family members. Supplemental nutrition assistance programs, specifically WIC and SNAP, provided critical assistance, although some expressed eligibility barriers. Some felt that without these services, their food insecurity would be unmanageable (quotations 38 and 39). Other mothers noted difficulty obtaining services (quotation 40) or the funds not lasting the whole month (quotation 41).	Semistructured qualitative interviews revealed that supplemental nutrition assistance programs, specifically WIC and SNAP, provided critical assistance, although some expressed eligibility barriers. Some felt that without these services, their food insecurity would be unmanageable.
Coping strategies and nutrition education needs among food pantry users	Anne Hoisington (2002) [32]	The purposes of this study were to: *1*) identify effective coping strategies associated with stretching food resources that can provide a foundation for nutrition education; *2*) identify barriers to and limits of coping strategies in alleviating food scarcity that may need to be addressed in nutrition education, and *3*) determine channels of nutrition education that would help families with coping strategies.	Cross-sectional study, qualitative methods; Radimer/Cornell	Food pantry users in 9 locations across Washington State. The mean age was 34 y, with a range of 16–56 y. Most of the participants were female (69%) and White (79%).	Teens, mothers, fathers	Making food in bulk; using up leftovers; making use of what was on hand; freezing foods for later use; participants used a combination of stores, discount coupons, and sales to obtain foods and ingredients; numerous participants were creative in mimicking popular convenience foods by using bulk ingredients to cut costs; food substitutions were common and included substituting powdered milk for fresh, canned or frozen vegetables for fresh, dried beans for canned, and cheaper cuts of meats for more expensive ones, reducing or omitting unaffordable ingredients such as meat was common, as was increasing the amount of inexpensive and filling ingredients such as potatoes or noodles; certain ways of ensuring that there was enough food could put families at risk, including getting cash advances, putting off paying other bills, and cutting back on nonfood grocery items such as paper goods; making a choice between food and other needed supplies or services was a common element of coping; 1 participant said her family gathered “roadkill” when it was available; food insecurity pushed families to look for atypical sources of food, such as emergency foods, shared meals with others, trading labor for food, and trading food to diversify food resources; in 1 rural location, domestic food production activities were critical in providing food and included canning or preserving homegrown foods, hunting and fishing, raising meat, and food gathering or foraging	Participants described diverse coping strategies that were highly integrated and creative, but that did not ultimately stave off hunger in many cases. Although the progressive nature of hunger and coping strategies was not investigated directly, it appeared that people used increasingly more desperate coping strategies as food became scarcer and other problems became more apparent in the household. For example, the end of the month is an especially difficult time to provide nutritious foods for the family. One participant explained, “Well, I’ve got 3 teenagers to feed, and they eat like horses, so it’s kind of like feast and famine around our house. When the food stamps show up it’s feast, and then toward the end of the month it’s macaroni and cheese famine.”	Not applicable
Food and financial coping strategies during the monthly Supplemental Nutrition Assistance Program cycle	Eliza Whiteman Kinsey (2019) [33]	The purpose of this study was to provide a critical exploration of the nature and timing of coping strategies for managing the SNAP cycle, including implications these coping mechanisms have for health and financial stability.	Longitudinal cohort study, qualitative methods; 18-Question United States Household Food-Security Survey Module	Mothers (*n =* 12) receiving SNAP benefits in Philadelphia between 2016 and 17. Mean age was 34.8, and participants had a mean BMI of 32.8 kg/m^2^. The majority of participants were single, and the mean household size was 3.8 people. Two-thirds of participants reported very low household food security (compared with 39% of food-insecure households nationally).	Mothers	Adjustments to food shopping and eating; eating less expensive, energy-dense (and often less healthy) foods to fill up; skipping meals; mental accounting and resilience strategies; social support strategies; delaying bills or paying minimum amount; rationing SNAP for multiple shopping trips; borrowing money and/or food from friends and family	Participants shared numerous deliberate food shopping and eating strategies for managing the end-of-month period. A key finding from our study is the different types of shopping trips participants made depending on the timing within the SNAP cycle. For example, the first shopping trip after receiving SNAP benefits was typically for stocking up on essential items, such as meats and proteins, fruits, vegetables, and grains. Many of the women (58.3%) reported creating a weekly or monthly food budget for their household. Participants frequently highlighted the importance of social networks in mitigating food and financial insecurity. They referenced a range of assistance, reflecting instrumental, emotional, and informational support.	Semistructured interviews revealed that the coping strategies households use for managing the SNAP cycle have short-term benefits, such as buffering against hunger. However, these coping strategies include making tradeoffs that often compromise health and may have long-term negative financial repercussions.
The psychological distress of food insecurity: a qualitative study of the emotional experiences of parents and their coping strategies	Cindy W. Leung (2022) [34]	The purpose of this study was to understand parental experiences of psychological distress specific to food insecurity, with a specific focus on identifying types of psychological distress, experiential descriptions, and the array of emotional responses and coping strategies specific to food insecurity.	Phenomenological study, qualitative methods; 18-Question United States Household Food-Security Survey Module	Forty-eight adults (parents) with a child aged 7–14 y were recruited from the San Francisco Bay Area in 2016–2017 with any experience of household food insecurity over the past 12 mo. There were 43 mothers and 5 fathers (mean age = 36.4 y). The racial/ethnic distribution of the parents was as follows: 29% White, 29% Black, 2% Asian, 25% Hispanic/Latino, and 15% as another racial/ethnic category or multiracial. This last race/ethnicity category included participants who identified as Native American, Pacific Islander, and of ≥2 races/ethnicities. Approximately 44% of parents were single or never married, 40% were married or living with a partner, and 17% were divorced. The majority of parents (63%) were currently working for pay. With respect to food-security status (assessed during the screening interview), 8% of families had marginal food security, 42% had low food security, and 50% had very low food security.	Mothers, fathers	Parents resorted to highly processed foods (e.g., instant noodles, frozen lasagna, or hot dogs) that were cheaper and readily available in their neighborhoods; couldn't afford to buy fresh vegetables; utilized food assistance, such as food stamps or local food pantries; relied on friends and family for support; coped with food insecurity by avoiding interaction with others, sleeping, drinking, and spent more time with their children	Parents expressed negative and positive coping responses for the distress they felt related to food insecurity. Negative responses included avoiding interaction with others, sleeping, and drinking. Positive coping responses included relying on friends and family for support, seeing a mental health professional, praying, and staying optimistic.	Not applicable
Understanding the psychological distress of food insecurity: a qualitative study of children’s experiences and related coping strategies	Cindy W. Leung (2020) [35]	The purpose of this study was to obtain a better understanding of the psychological distress of food insecurity in children to lend insight into the underlying mechanisms between food insecurity and children’s health outcomes. The authors use the broader term psychological distress to refer to the overall negative affectivity that may result from food insecurity, and distinguish this from stress, which often refers to a physiological, behavioral, or biochemical reaction in response to an uncomfortable event.	Cross-sectional study, qualitative methods; 18-Question United States Household Food-Security Survey Module	Children (7–14 y) from the San Francisco Bay Area whose parents reported any experience of household food insecurity over the past year. There were 32 boys and 28 girls included. Children’s ages were as follows: 19 children were aged 7–8, 23 were aged 9–11, and 10 children were aged 13–14. Children represented 48 families, of which 16 had >1 child participate in the study. Twenty-nine children identified as White, 16 were Black, 4 were Asian or Pacific Islander, 5 were Native American, and 6 were multiracial. Of these, 16 children identified as Hispanic. Using parents’ reports of household food security, 8% of families had marginal food security, 43% had low food security, and 50% had very low food security.	Children, teens	Distracting from or using imagination to cope with food insecurity; Increasing tolerance of the family’s food situation; appreciating parents for providing food and resources	Most children expressed awareness of their family’s food insecurity and of insufficient food resources in the home. In addition to general awareness, children were also aware of how their extended family provided them with food and money in times of hardship, and that their family used food assistance programs (e.g., free school meals, SNAP benefits) or food pantries to help make ends meet. Children then discussed their experience of psychological distress related to food insecurity and strategies they employed to tolerate or cope with food insecurity. Children described several themes pertaining to how they coped with or adapted to food insecurity: distracting from or using imagination to cope with food insecurity, increasing tolerance of their family’s food situation, and appreciating their parents for providing food and resources.	Not applicable
Understanding the relationship between food security and mental health for food-insecure mothers in Virginia	Rachel A. Liebe (2022) [24]	The purpose of this study was to understand the differences in the relationship between mental health and food insecurity among mothers with low and very low food security (i.e., high and very high food insecurity) and to elucidate the potential factors, including physical health, social support, and food coping strategies, influencing this relationship.	Cross-sectional study, quantitative methods; 18-Question United States Household Food-Security Survey Module	Mothers with low income in Virginia who reported being food insecure. All respondents were food insecure; 232 respondents were experiencing low food security, and 797 were experiencing very low food security. Respondents ranged in age from 18 to 80 y old and were living in a household with 1–9 children. Approximately two-thirds of respondents reported living with a spouse or unmarried partner. Household incomes ranged from USD 0 to 80,000. Respondents reported participating in 2.0 ± 1.3 assistance programs in the past year, with over half of respondents reporting that they received SNAP benefits. Overall, 65.0% (*n =* 673) of respondents identified as White, 23.4% (242) as Black or African American, and 9.7% (100) as Hispanic/Latino. More than three-quarters of respondents reported their educational attainment as some college or less.	Mothers	Use of assistance programs (SNAP, WIC, National School Lunch Program/School Breakfast Program, Head Start, Temporary Assistance for Needy Families, Food Banks, The Emergency Food Assistance Program, Public Housing, USDA Summer Meals, SNAP-Ed); use of social support; tradeoffs (between medical, utilities, rent/mortgage, transportation, and education); financial (asked friends and family for food or money for food; sold food or pawned any personal property; skipped paying bills to buy food; bought the cheapest food available; avoided buying expensive foods, for example, fruits/vegetables); rationing (locked up or hid food to save it; stretched food by limiting; avoided having guests to avoid serving food; eaten as much as possible when food is available; eaten meals or snacks after children finished), visited a social or community event just to eat; removed spoiled parts from fruits/vegetables	Increased use of food coping strategies was positively correlated with increased anxiety (ranged from 0.40 to 0.41) and depression symptoms (0.39–0.43; *P* < 0.001). Respondents with very low food security used more food coping strategies than those with low food security (ranging from −0.9 to −1.5 for the types of food coping strategies, *P* < 0.001).	Not applicable
Differing within-household food-security statuses are associated with varied maternal mental health outcomes	Rachel A. Liebe (2024) [23]	The purpose of this study was to explore how the food-security status of both adults and children in the household is associated with maternal mental health. Understanding this association can be used to identify ways mothers successfully cope with limited resources and identify opportunities for public health practitioners to adapt interventions that address food insecurity to further support coping strategies.	Cross-sectional study, quantitative methods; 6-Question Short Form United States Household Food-Security Survey Module	Virginia mothers with low income (August–October 2021) were included. There were 570 respondents who met the eligibility criteria, of which 55.6% (317) were classified as living in a reference household, 39.5% (225) as adult-only food-insecure households, and 4.9% (28) were child-only food-insecure households. Respondents ranged from 18 to 80 y old and reported living with 1–7 children. Children’s age ranged from <1 mo to 17 y old. Respondents primarily identified as White (63.0%) or Black or African American (25.3%). Reported household income ranged from USD 0 to 80,000. Across all groups, 59.1% of respondents reported participating in SNAP.	Mothers	Behavioral food coping strategies; tradeoffs; financial, rationing; relying on social support	Mothers in the child-only food-insecure and adult-only food-insecure groups used 3.1 and 4.0, respectively, more behavioral food coping strategies than mothers in reference households (*P* < 0.001). SNAP participation was associated with a small increase in behavioral food coping strategy usage (0.5, *P* < 0.05) and with reduced social support (−1.2, *P* < 0.05).	The results of this cross-sectional survey revealed that, based on the percentage of the full sample that experienced maternal but not child food insecurity, mothers appear to be shielding their children from experiencing food insecurity, which is in line with other research. They reported using more behavioral food coping strategies than mothers in reference households. However, the act of shielding children may be impacting their mental and physical health.
“I’m doing the best that I can”: mothers lived experience with food insecurity, coping strategies and mental health implications	Rachel A. Liebe (2024) [36]	The purpose of this study was to understand the perspective of Virginia mothers experiencing food insecurity on the stressors they face, the coping strategies they utilize, and the implications for their mental health.	Descriptive study, qualitative methods; 18-Question United States Household Food-Security Survey Module	Semistructured interviews were conducted in May and June 2022 with a purposive sample of Virginia mothers who reported experiences of food insecurity. Mothers (*n =* 15) predominately identified as White and not Hispanic or Latino (80.0%, *n =* 12), lived in a household experiencing very low food security (66.6%, *n =* 10), and reported a score of 48.8 (range 25.8–64.6) on the Patient-Reported Outcomes Measurement Information System Global Mental 2a Scale., with a higher score indicating better mental health and a score of 50.0 being common for the United States population.	Mothers	Mothers engage in multiple coping strategies to alleviate stress caused by food insecurity; use available social and/or community resources; engage in actions to alter financial status; reframe shopping expectations; reduce personal food intake; implement avoidance behaviors	The findings of this study suggest that mothers use multiple coping strategies to alleviate the stress of food insecurity. This is consistent with existing literature suggesting households often have to rely on multiple strategies, especially when household resources are unstable, which may have been the case in this study, given the economic context described earlier.	Not applicable
Generations of “shock absorbers”: women caregivers of young children and their efforts to mitigate food insecurity during the COVID-19 pandemic	R. Lindberg (2024) [37]	The purpose of this study was to answer the research questions: *1*) How has food insecurity affected the childhood, current life, and the children in the care of low-income caregivers? *2*) How effective were the special pandemic measures to address the shocks to the food system for this population in 2 varying contexts and countries? and *3*) What vulnerabilities are evident in policies and programs that aim to support food security in households with young children and what can be improved to ensure sustained food security?	Descriptive case study, qualitative methods; a validated 2-item household food-security screening tool was included within the interview to provide a concise assessment of food-security status	This study investigated food insecurity from the perspective of caregivers in Australia and the United States, especially their perceptions of the impact of food insecurity on their own childhood, their current life, and the children in their care. A majority (63%) of participants were homemakers/not in paid employment, and just over half were single caregivers (51%). Of the 41 participants, 76% were screened as food insecure, 68% had 3 or fewer children, and 29% were pregnant at the time of the interview.	Mothers, caregivers	Turning to emergency and community food sources; United States federal food assistance programs like SNAP and WIC became increasingly important; some caregivers relied on more informal support systems, such as family and neighbors to help watch the children, so they would have time to access food resources, and for social support; the food families received from food pantries was not always suitable and these caregivers felt obliged to not let food go to waste; interviewees set rules around food; other food coping strategies included caregivers eating last or not at all; reducing meat intake; hiding food; utilizing coping strategies replicated from their own childhood; participants described fussy eaters or older children were often given priority; caregivers explained that they tried to reduce nagging at the shops and advise children before outings that there were limits on what they could expect to receive as treats	At the time of the interviews, three-quarters of the participants were screened as food insecure, and just over half were single caregivers. Six themes were established, including: *1*) growing up poor; *2*) lessons learned; *3*) feeding a family amidst a pandemic; *4*) caregiver coping strategies; *5*) food-security challenges in the early years, and *6*) protecting (young) children.	Not applicable
Prevalence of food insecurity in low-income neighborhoods in West Texas	Mary W. Murimi (2016) [38]	The purpose of this study was to determine the prevalence and correlates of food insecurity and related coping strategies among African American and Hispanic households in urban and rural West Texas.	Cross-sectional study, quantitative methods; 18-Question United States Household Food-Security Survey Module	Sample size was 191 participants from low-income households, predominantly African American and Hispanic people. A total of 191 individuals participated in this study. Fifty-eight participants came from rural households, and 133 came from urban households in West Texas. Hispanic and African American individuals made up 67% and 33% of the total sample, respectively. Rural respondents were predominantly Hispanic (95%), whereas urban respondents were made up of almost equal proportions of African American (46%) and Hispanic people (47%). Rural respondents were more likely to have less than a high school diploma compared with urban respondents (33% and 7%, respectively*; P* < 0.001). However, a greater proportion of urban respondents reported making below $25,000/y compared with rural respondents (66% and 51%, respectively; *P* = 0.04)	Mothers, fathers	Reducing portions per meal; eating more energy-dense food because it was affordable; skipping meals; participation of households in food and nutrition assistance programs; food pantry or food bank; soup kitchen; senior meal sites; relying on a few kinds of low-cost food to feed their children	Participants in this study indicated that they were coping with the challenges of food insecurity mainly by reducing portions per meal, eating more energy-dense food because it was affordable, and skipping meals in some cases. Significantly more members of food-insecure urban households compared with food-insecure rural households reported cutting the size of meals or skipping meals (61% vs. 25%; *P* = 0.02), eating less than they felt they should (68.5% vs. 29%; *P* < 0.001), or going hungry (55% vs. 13%; *P* < 0.001), respectively. Approximately 29% of food-insecure urban people reported losing weight (*P* = 0.03) or not eating for the whole day (*P* = 0.01) compared with only 6% of food-insecure rural respondents. A majority of both rural and urban food-insecure households (87%) reported relying on a few kinds of low-cost food to feed their children, compared with 8% of food-secure households. Both rural and urban food-insecure households (69%) reported that they could not feed their children balanced meals, compared with none of the food-secure households. Participants from urban households reported that their children were not eating enough (58%; *P* = 0.01) and experienced hunger (26%; *P* = 0.03). Urban households were more likely to participate in SNAP than were rural households (51% vs. 17%; *P* < 0.001), whereas rural households were more likely to participate in the Emergency Food Assistance Program than were urban households (31% vs. 2%; *P* < 0.001).	Not applicable
Children’s reporting of food insecurity in predominantly food-insecure households in Texas Border Colonias	Courtney C. Nalty (2013) [39]	The purpose of this study was to answer the primary research question: How do intrahousehold mother and child reports of food security differ according to questions of the 18-item Household Food-Security Survey Module and the 9-item Food-Security Survey Module for Youth? Secondarily, using the 8 child-referenced items of the Household Food-Security Survey Module, how does mother-reported child food security contrast with child-reports of food security when children report using the Food-Security Survey Module for Youth?	Cross-sectional study, qualitative methods; 18-Question United States Household Food-Security Survey Module	Food-security discordance was evaluated among 50 Mexican-origin children ages 6–11 and their mothers living in Texas border colonias from March to June 2010. Median age (SD) of mothers was 34.5 y (± 6.9), whereas child median age was 8.5 y (± 1.3). Ninety-two percent of mothers were born in Mexico, with the remainder born in the United States, and mothers completed a median 9 y (± 2.5) of school. Median household size was 6 adults and children (± 1.5, range: 3–10), and median number of children living in the home was 3.5 (± 1.2, range: 1–6). In this sample, 96% of children were enrolled in the School Breakfast Program, 88% of families used SNAP, 58% of families relied on WIC, 58% of children were enrolled in the National School Lunch Program, and 32% of families were enrolled in all 4 nutrition assistance programs.	Mothers, children	Low-cost foods for children; adults reduce children’s portion sizes; child skips meals	Coping strategies were not discussed as an outcome in relation to the study objectives.	Not applicable
Household food security among migrant and seasonal Latino Farmworkers in North Carolina	Sara A. Quandt (2004) [40]	The purpose of this study was threefold: to characterize levels of food security, food insecurity, and hunger among migrant and seasonal Latino farmworkers; to assess predictors of food insecurity for this group; and to describe the strategies farmworkers use to cope with food insecurity.	Cross-sectional study, qualitative methods; Spanish-language adaptation of the 18-item United States Household Food-Security Survey Module	Adults from 102 farmworker households in North Carolina responded to a survey that used a Spanish-language adaptation of the United States Household Food-Security Survey Module and questions about sociodemographic characteristics and food behaviors. Forty-eight of the 102 sample households (47.1%) were classified as food insecure, including 10 (9.8%) with moderate hunger and 5 (4.9%) with severe hunger. Households with children had a significantly higher prevalence of food insecurity than those without children (56.4% vs. 36.2%).	Mothers, fathers	Stretching food dollars; utilizing local church food pantries and food distribution programs run by social service organization; supplement their food with wild game and fish; informal borrowing arrangements; government food programs; planning ahead	Households with children accessed food programs such as WIC that were unavailable to those without children, while those without children were more likely to access food pantries and to consume wild game or fish. Coping strategies included borrowing money, reducing food variety, and adults consuming less food to protect children from hunger.	From questionnaire data collected through interviews, researchers found that the use of wild game was associated with food security in households with children.
Remembering food insecurity: low-income parents’ perspectives on childhood experiences and implications for measurement	Tracey L. Rosa (2018) [41]	The purpose of this study was to explore how a diverse group of low-income parents recalled and described childhood experiences with food insecurity, to understand how these experiences were perceived later in life, and to inform the development of measurement approaches to identify other parents who had such experiences.	Retrospective study, qualitative methods; 18-Question United States Household Food-Security Survey Module	A diverse group of 27 low-income mothers in New York State was interviewed in depth. All interviewees were women, and most were the primary caregiver of ≥1 child between the ages of 2 and 12; 3 had older children, and 2 were fostering grandparents. Almost all had participated in food-related and other social service programs.	Children, mothers	Strategies to increase household access to food; strategies during preparation and mealtimes to make food available to children; strategies for making available meals more acceptable and enjoyable; stocking up on food and buying on sale; going to food pantries; gleaning; stealing; asking family or neighbors for assistance; family members working more to earn extra income; meal planning; using less expensive foods as alternatives to pricier choices; stretching meals; some participants reported that parents took smaller portions or skipped meals, prioritizing children’s food intake over theirs; the types of dishes their parents used to try to stretch food included soups, stews, roasts, chopped meat, meatloaf, and pasta dishes; participants remembered their mothers or grandmothers cooking in big batches and making sure there were leftovers, and adding foods to a leftover dish so it would feed everyone; several participants spoke about strategies their parents used to break up monotony in meals or make children feel like they were getting a special treat; in these cases, parents tried to protect their children not only from hunger, but also from feeling deprived or unhappy with available foods; most commonly, participants talked about their parents adding variety to their meals; sitting down together to enjoy mealtime as a family was also mentioned; parents also added desserts or sweets to a meal, as a way to “make a plain meal grand”	Key emergent themes were: strong, negative emotions elicited by memories of food insecurity; protective instincts when recalling parents’ efforts to ensure that children had something to eat; variation in awareness of economic causes of food insecurity; and emphasis on family strategies for coping with or avoiding food insecurity.	During cognitive interviews, many participants spontaneously described strategies their families used to limit food insecurity and provide satisfying meals. Participants often seemed more comfortable discussing these strategies and solutions than the problems. Our analysis identified 3 categories of strategies: strategies to increase the amount of food coming into the household, despite limited resources; strategies used during preparation and mealtimes to ensure that children always had something to eat; and preparation/mealtime strategies to make available foods and meals more acceptable and desirable for children.
Child Hunger and the Protective Effects of Supplemental Nutrition Assistance Program (SNAP) and alternative food sources among Mexican-origin families in Texas Border Colonias	Joseph R Sharkey (2013) [42]	The purpose of this study was to examine child hunger among 470 Mexican-origin families by: *1*) determining the prevalence of child hunger, and *2*) identifying protective and risk factors associated with hunger among children using data from the 2009 Colonia Household and Community Food Resource Assessment (C-HCFRA).	Cross-sectional study, qualitative methods; Radimer/Cornell	Data included all 470 participants in the 2009 Colonia Household and Community Food Resource Assessment in the Texas border region who reported that ≥1 child under the age of 18 y resided in the home. Almost 51 percent reported child hunger—their “child(ren) is(are) hungry sometimes, but [parents/caregivers] can’t afford more food.” Sixteen percent of respondents who reported no child hunger indicated adult hunger, and 41.3% (*n =* 194) of households reported no adult or childhood hunger.	Caregivers	Grocery purchases (use of a supermarket, supercenter, or dollar store); use of nutrition assistance programs (SNAP, WIC, School Breakfast Program, National School Lunch Program, Emergency); local food environment (little variety, few grocery stores, high prices); food challenges (no balance during school year or no balance during summer); use of alternative food sources (neighbor/friend, Mobile Food Vendor, pulga (flea market))	A smaller percentage of households with child hunger participated in school-based nutrition programs (51%) or used alternative food sources, whereas 131 households were unable to give their child or children a balanced meal during the school year, and 145 households during summer months. In the random effects model (RE = small town), increased household composition, full-time unemployment, and participation in the National School Lunch Program were significantly associated with increased odds for child hunger, whereas participation in SNAP and purchasing food from a neighbor were significantly associated with decreased odds for child hunger.	The survey results indicated that among community resources, participation in SNAP and buying food from a neighbor or friend were associated with significantly reduced odds for child hunger by 53% and 49%, respectively.
Dietary intake, overweight status, and perceptions of food insecurity among homeless Minnesotan youth	Chery Smith (2008) [43]	The purpose of this study was to investigate how youth living in urban homeless shelters in Midwestern America cope in an adverse food environment, and how it impacts their dietary intake and body mass index status.	Cross-sectional study, mixed methods; USDA’s instrument on food security for adults was modified and the youth were asked to respond to the following 4 statements: *1*) ‘‘There are times when we do not have enough food in the house,” *2*) ‘‘I go to bed hungry at night,” *3*) ‘‘I do not get enough to eat at home,” and *4*) ‘‘Have you ever had to miss a meal (or not been able to eat) because there was no food at home?’’	Youth, 9–18 y (*n =* 202; 106 females and 96 males), living in homeless shelters in Minneapolis, Minnesota, were participants. All youth lived in homeless shelters with ≥1 parent, and the mean household size was 4.8 ± 1.8 people, with 3.4 ± 1.6 children. Youth lived in the shelter for varying lengths of time (range: 3 d to 12 mo at their present shelter), and many said that they had lived in shelters before this time, though that was not recorded.	Children, teens	Overeat at mealtimes to not be hungry later on; if hungry, will eat anything; if hungry, will eat foods that they do not like; when am hungry, eat snacks like chips, candy, and pop; when there is not a lot of food in the home, eat at a friend’s or family’s house; if there is no food at home, find food somewhere else	Overeating, eating anything, eating disliked foods, and eating at the homes of family and friends were identified as strategies to cope with food insecurity. Overeating when food is available may explain why a hunger-obesity paradigm is observed to the magnitude seen among the poorest Americans. These strategies protect children from the immediate negative associations of poverty and hunger, but they may contribute to long-term weight problems currently found in the United States.	Not applicable
Exploring food insecurity among young mothers (15–24 y)	Christine A. Stevens (2010) [44]	The purposes of this study were to: *1*) describe the prevalence of food insecurity in these mothers; *2*) identify factors that contribute to food insecurity, and *3*) describe strategies that young mothers employ to plan for and manage food insecurity.	Descriptive study, qualitative methods; 18-Question United States Household Food Security Survey Module	Participants were young mothers (15–24 y) who are single heads of households with children. Results from the indicated that 76% (*n =* 16) of participants had food insecurity. Eleven participants reported low food security (reports of reduced quality or desirability of diet), and 5 reported very low food security (reports of disrupted eating patterns and reduced food intake). Eighty-five percent (*n =* 18) of the young mothers received external assistance such as WIC, Temporary Assistance to Needy Families, and food stamps.	Mothers	Skipping meals in order for the children to eat; cutting meals so the children can eat; planning ahead so children could eat by buying food with long shelf life; supplement monthly food with food banks; employing multiple approaches; trying to budget; using external federal sources; using family members to supplement their incomes; using cereal in baby’s milk to add bulk to feedings and help with hunger	All the strategies used by the young mothers had one goal, which was to make sure that their children always had food. Overall, the young women all discussed what they considered to be good nutrition for themselves and their children. Many of them took advantage of nutrition classes at WIC or were aware of the food pyramid and struggled to address those needs. The social factors of housing, income, food cost, and access influenced their abilities to plan and provide nutritious meals.	During semistructured interviews, all the women reported that their children had only marginal food insecurity due to extensive strategies.
Examining factors related to the food insecurity–obesity paradox in low-income mothers and fathers	Emily A. Taylor (2021) [45]	The purpose of this study was to examine factors that may be related to the food insecurity–obesity paradox within a sample of low-income mothers and fathers. The objectives were to determine: *1*) whether relationships exist between food insecurity, depression scores, and BMI in this sample of mothers and fathers of young children, and *2*) differences by gender in reports of parents’ sacrificing their own diet to feed their children.	Cross-sectional study, mixed methods; 18-Question United States Household Food-Security Survey Module	Participants were low-income cohabiting mother and father pairs (*n =* 25) living with their child. The majority (58%) of the sample was overweight or obese (76%) and White (58%).	Mothers, fathers	Parents reported sacrificing food for children; prioritizing; parents also mentioned eating something else if there was not enough of the meal to go around	There was a significant (*P* = 0.003) difference in report of adults in the household sacrificing consumption to feed young children between mothers (2.91 ± 0.92) and fathers (3.59 ± 0.73), with mothers reporting greater sacrifice and compromised diet quality to feed their children, but no significant correlation among BMI, depression, and food insecurity was detected.	Not applicable
Household food insecurity and dietary patterns in rural and urban American Indian Families with young children	Emily J. Tomayko (2017) [46]	The purpose of this study was to address 3 main research questions: *1*) What is the prevalence of food insecurity in American Indian households, and how did this differ by urban-rural status? *2*) What are the correlates of food insecurity in American Indian households? and *3*) What is the relationship between food insecurity and diet in these households?	Cross-sectional study, mixed methods; 18-Question United States Household Food-Security Survey Module	Dyads consisting of an adult caregiver and a child (2–5 y old) from the same household in 5 urban and rural American Indian communities were included. In total, 450 adult-child dyads from 5 participating communities (*n =* 240 rural households; *n =* 210 urban households) were enrolled. For adults, the mean age was 31.5 ± 8.5 y, 95% were female, and 81.3% self-identified as American Indian; for children, the mean age was 45.0 ± 13.0 mo, 50.0% were female, and 86.3% were identified by their caregiver as American Indian. The overall prevalence of food insecurity was 61% and was significantly higher in urban vs. rural households at 80% vs. 45%, respectively (*P* < 0.001).	Mothers, fathers, caregivers	Factors associated with shopping; family sharing practices; use of food assistance programs; other coping strategies (i.e., “We buy stuff we know will keep, like boxes of cereal and pasta, like when you can get 10 boxes of noodles for $10.”); reliance on local produce or bartering (rural only); cost, perceived value, and time (related to food choice)	Participants reported coping strategies employed during times of food insecurity, such as use of food assistance programs and relying on family members to supplement meals. Participants reported some intergenerational living or childcare arrangements, which blunted some food insecurity through pooled resources but introduced a loss of parental control over some feeding choices. Some geographic differences in coping strategies were noted. For example, urban families with greater access to food outlets reported shopping frequently (every day or every other day), which resulted in spending more on food than planned. Rural families reported infrequent food purchasing trips, which often resulted in the purchase of fewer fresh fruits and vegetables. Rural families also reported using hunting, gathering, and sharing practices (e.g., hunting deer, harvesting wild rice) and individual/community gardens to supplement their diet.	Not applicable
Livelihood strategies of food-insecure poor, female-headed families in rural Alabama	Andrew A. Zekeri (2007) [47]	The purpose of this study was to examine how food-insecure, poor, single mothers commonly get food for themselves and their children. There is little systematic information by region and nation that depicts how food-insecure single mothers get their food, and little is known about their other livelihood strategies in a rural context.	Cross-sectional study, qualitative methods; 6-Question Short Form United States Household Food-Security Survey Module	100 African American single mothers from rural Alabama were recruited. Majority (85.7%) were African Americans, and 44.3% had no education beyond high school. Fifty-seven percent earned <$10,000, and 13.3% earned between $10,000 and $14,000. Forty-nine percent were unemployed, and 55.9% were not receiving food stamps.	Mothers	Employment; government assistance; informal support; cash assistance from absent fathers; cash assistance from boyfriends; cash assistance from relatives; community groups, churches, and social agencies, food banks, churches; cohabiting; doubling-up; eating at a senior meal program; belt-tightening (making cheaper and smaller meals)	The findings show that most of the mothers used numerous strategies to make sure that there was an adequate amount of food for the family. These strategies included work, government assistance such as food stamps, cash assistance from relatives and friends, food from food banks and churches, cohabiting, coresiding with a friend or relative, eating at a Senior Meal Program, and eating less.	Not applicable

Abbreviations: SNAP, Supplemental Nutrition Assistance Program; WIC, Special Supplemental Nutrition Program for Women, Infants, and Children.

## Results

A total of 25 observational studies were included, using qualitative (*n* = 17, 68%), quantitative (*n* = 4, 16%), and mixed methods (*n* = 4, 16%). These studies reported a range of designs, including cross-sectional (*n* = 19, 76%), descriptive (*n* = 2, 8%), case study (*n* = 1, 4%), phenomenological (*n* = 1, 4%), retrospective (*n* = 1, 4%), and longitudinal cohort (*n* = 1, 4%). As described in Table 2, most studies collected information on the use of coping strategies by mothers (*n* = 19, 47.5%). Fewer studies explored the use of techniques by fathers (*n* = 7, 17.5%), caregivers (*n* = 4, 10%), teens (*n* = 5, 12.5%), and children (*n* = 5, 12.5%). Thirteen studies reported the use of coping strategies by multiple members of a household.

**TABLE 2:** Coping strategies used to maintain food security by mothers, fathers, caregivers, children, teens, and multiple household members in 25 studies exploring coping strategies used by United States households with children to maintain food security.

Title	First author	Mothers	Fathers	Caregivers	Children	Teens
Working to eat: vulnerability, food insecurity, and obesity among migrant and seasonal farmworker families	Kristen Borre [26]	X	X			
Stretching food and being creative: caregiver responses to child food insecurity	Michael P. Burke [27]			X		
Teen food insecurity: finding solutions through the voices of teens	Mecca Burris [28]					X
Children are aware of food insecurity and take responsibility for managing food resources	Maryah Stella Fram [22]				X	X
Questionnaire-based measures are valid for the identification of rural households with hunger and food insecurity	Edward A. Frongillo, Jr. [29]	X				
Maternal strategies to access food differ by food-security status	Kathleen S. Gorman [30]	X				
Food insecurity during pregnancy and breastfeeding by low-income Hispanic mothers	Rachel S. Gross [31]	X				
Coping strategies and nutrition education needs among food pantry users	Anne Hoisington [32]	X	X			X
Food and financial coping strategies during the monthly SNAP cycle	Eliza Whiteman Kinsey [33]	X				
The psychological distress of food insecurity: a qualitative study of the emotional experiences of parents and their coping strategies	Cindy W. Leung [34]	X	X			
Understanding the psychological distress of food insecurity: a qualitative study of children’s experiences and related coping strategies	Cindy W. Leung [35]				X	X
Understanding the relationship between food security and mental health for food-insecure mothers in Virginia	Rachel A. Liebe [24]	X				
Differing within-household food-security statuses are associated with varied maternal mental health outcomes	Rachel A. Liebe [23]	X				
“I’m doing the best that I can”: mothers lived experience with food insecurity, coping strategies and mental health implications	Rachel A. Liebe [36]	X				
Generations of “shock absorbers”: women caregivers of young children and their efforts to mitigate food insecurity during the COVID-19 pandemic	R. Lindberg [37]	X		X		
Prevalence of food insecurity in low-income neighborhoods in West Texas	Mary W. Murimi [38]	X	X			
Children’s reporting of food insecurity in predominately food-insecure households in Texas Border Colonias	Courtney C. Nalty [39]	X			X	
Household food security among migrant and seasonal Latino farmworkers in North Carolina	Sara A. Quandt [40]	X	X			
Remembering food insecurity: low-income parents’ perspectives on childhood experiences and implications for measurement	Tracey L. Rosa [41]	X			X	
Child hunger and the protective effects of SNAP and alternative food sources among Mexican-origin families in Texas border Colonias	Joseph R Sharkey [42]			X		
Dietary intake, overweight status, and perceptions of food insecurity among homeless Minnesotan youth	Chery Smith [43]				X	X
Exploring food insecurity among young mothers (15–24 y)	Christine A. Stevens [44]	X				
Examining factors related to the food insecurity–obesity paradox in low-income mothers and fathers	Emily A. Taylor [45]	X	X			
Household food insecurity and dietary patterns in rural and urban American Indian Families with young children	Emily J. Tomayko [46]	X	X	X		
Livelihood strategies of food-insecure poor, female-headed families in rural Alabama	Andrew A. Zekeri [47]	X				

Abbreviation: SNAP, Supplemental Nutrition Assistance Program

Across the included studies, a total of 82 distinct coping strategies were identified. As presented in Table 3, the coping techniques were consolidated into 5 major themes: *1*) food acquisition strategies, *2*) food management strategies, *3*) financial coping strategies, *4*) relying on support from family members or friends, and *5*) children implementing strategies to help maintain food-security status for the household.

**TABLE 3:** Appearance of 82 distinct coping strategies implemented in United States households with children, grouped into 5 major themes across 25 studies exploring coping strategies used by United States households with children to maintain food security

Distinct coping strategy identified	Appeared in study	Coping strategy subtheme	Coping strategy theme
Budgeting while shopping for food (using coupons, buying store brands, etc.)	Borre (2010) [26], Burke (2017) [27], Gorman (2017) [30], Hoisington (2002) [32], Kinsey (2019) [33], Liebe (2024), Stevens (2010) [44]	Shopping or purchasing techniques	Food acquisition strategies
Purchasing low-cost foods	Borre (2010) [26], Burke (2017) [27], Gorman (2017) [30], Gross (2019) [31], Hoisington (2002) [32], Kinsey (2019) [33], Leung (2022) [34], Liebe (2022) [24], Murimi (2016) [38], Quandt (2004) [40], Rosa (2018) [41], Tomayko (2017) [46]		
Changes in foods purchased or obtained for household meals	Burke (2017) [27], Hoisington (2002) [32], Kinsey (2019) [33], Quandt (2004) [40]		
Cut quantity and quality of meats	Burke (2017) [27], Hoisington (2002) [32], Lindberg (2024) [37]		
Cut some foods (exact foods not specified)	Burke (2017) [27], Hoisington (2002) [32]		
Shop at budget grocery stores (e.g., Walmart, Aldi, Save-a-Lot)	Burke (2017) [27], Gorman (2017) [30], Liebe (2024), Quandt (2004) [40], Sharkey (2013) [42]		
Buy foods in bulk, especially meats	Burke (2017) [27], Gorman (2017) [30], Hoisington (2017) [32], Kinsey (2019) [33], Liebe (2024)		
Plan meals each week	Burke (2017) [27], Gross (2019) [31], Kinsey (2019) [33], Rosa (2018) [41], Stevens (2010) [44]		
Spent low amount on food and no money on eating out	Frongillo (1997) [29]		
Purchasing less junk food	Gorman (2017) [30]		
Shopping at multiple stores to get best deals	Gorman (2017) [30], Hoisington (2002) [32], Kinsey (2019) [33], Tomayko (2017) [46]		
Utilize a shopping list	Gorman (2017) [30], Gross (2019) [31], Kinsey (2019) [33]		
Purchasing fewer fruits and vegetables	Borre (2010) [26], Gorman (2017) [30], Kinsey (2019) [33], Leung (2022) [34], Liebe (2022) [24], Liebe (2024), Tomayko (2017) [46]		
Stocking up on food and necessities when there was extra money	Kinsey (2019) [33], Rosa (2018) [41]		
Stretching food dollars	Quandt (2004) [40]		
Eating at a senior meal program	Gorman (2017) [30], Zekeri (2007) [47]	Use of community assistance programs	
Visited a social or community event just to eat	Liebe (2022) [24]		
Relying on local churches or food banks for emergency foods	Borre (2010) [26], Burke (2017) [27], Frongillo (1997) [29], Gorman (2017) [30], Gross (2019) [31], Hoisington (2002) [32], Kinsey (2019) [33], Leung (2022) [34], Liebe (2022) [24], Liebe (2024), Lindberg (2024) [37], Murimi (2016) [38], Quandt (2004) [40], Rosa (2018) [41], Sharkey (2013) [42], Stevens (2010) [44], Tomayko (2017) [46], Zekeri (2007) [47]		
Participating in nutrition assistance programs (WIC, SNAP, HeadStart, National School Lunch Program, School Breakfast Program, etc.)	Borre (2010) [26], Burke (2017) [27], Frongillo (1997) [29], Gorman (2017) [30], Gross (2019) [31], Kinsey (2019) [33], Leung (2022) [34], Liebe (2022) [24], Liebe (2024) [23], Liebe (2024) [36], Lindberg (2024) [37], Murimi (2016) [38], Nalty (2013) [39], Quandt (2004) [40], Sharkey (2013) [42], Stevens (2010) [44], Zekeri (2007) [47]	Participating in nutrition assistance programs such as WIC, SNAP, HeadStart, National School Lunch Program, School Breakfast Program	
Migrant seasonal farmworkers reported taking foods from the field home or eating it, unwashed, in the field	Borre (2010) [26]	Utilizing alternative sources of food	
Garden to supplement food supply	Burke (2017) [27], Frongillo (1997) [29]		
Gathering roadkill when it is available	Hoisington (2002) [32]		
Hunting, fishing, or acquiring game to supplement food resources	Frongillo (1997) [29], Quandt (2004) [40]		
Relying on domestic food production (canning, foraging, raising meat, etc.)	Hoisington (2002) [32]		
Gleaning	Rosa (2018) [41]		
Use foods that can be stretched (e.g., stews, soups, casseroles, pasta, bean or rice dishes)	Burke (2017) [27], Gross (2019) [31], Hoisington (2002) [32], Murimi (2016) [38], Quandt (2004) [40], Rosa (2018) [41]	Strategies implemented during meal preparation	Food management strategies
Simplify meals	Burke (2017) [27]		
Use more canned goods	Burke (2017) [27], Hoisington (2002) [32], Kinsey (2019) [33], Stevens (2010) [44]		
Utilize shelf-stable and/or fast foods over fresh foods (e.g., boxed and canned dinners, dollar menus)	Burke (2017) [27], Frongillo (1997) [29], Gorman (2017) [30], Hoisington (2002) [32], Kinsey (2019) [33], Leung (2022) [34], Liebe (2022) [24], Stevens (2010) [44], Tomayko (2017) [46]		
Child food preferences come first	Burke (2017) [27], Leung (2022) [34]		
Changes to household meal patterns	Burke (2017) [27]		
Serve smaller portions at mealtime	Burke (2017) [27], Liebe (2022) [24], Murimi (2016) [38]		
Change mealtimes and frequency to stretch food	Burke (2017) [27]		
Have breakfast foods any time of day	Burke (2017) [27]		
Being creative, make stuff up or use what they have	Burke (2017) [27], Gross (2019) [31], Hoisington (2002) [32], Kinsey (2019) [33]		
Make use of leftovers and freeze meals	Burke (2017) [27], Gross (2019) [31], Hoisington (2002) [32], Kinsey (2019) [33], Quandt (2004) [40], Rosa (2018) [41]		
Cooking at home rather than eating out	Gross (2019) [31], Kinsey (2019) [33]		
Removed spoiled parts from fruits/vegetables	Liebe (2022) [24]		
Belt-tightening (making smaller meals)	Liebe (2022) [24], Zekeri (2007) [47]		
Child or teen relied on cheap and convenient foods (fast food, snacks, candy, etc.)	Burris (2020) [28], Smith (2008) [43]	Children implementing strategies that impact their personal intake	
Child skips meals	Leung (2020) [34], Nalty (2013) [39]		
Child eats as much as possible when food is available or overeats at mealtimes to limit hunger later on	Smith (2008) [43]		
If hungry, child will eat foods that are not preferred	Smith (2008) [43]		
Adults eat something else if there is not enough of the meal for them to eat	Taylor (2021) [45]	Adults implementing strategies that impact their personal intake	
Adults reduce their portions or do not eat at all	Burke (2017) [27], Kinsey (2019) [33], Leung (2020) [35], Liebe (2024), Lindberg (2024) [37], Murimi (2016) [38], Rosa (2018) [41], Stevens (2010) [44], Taylor (2021) [45]		
Adults eat as much as possible when food is available	Liebe (2022) [24]		
Adults prioritize children eating first	Liebe (2022) [24], Liebe (2024) [23], Liebe (2024) [36], Lindberg (2024) [37], Rosa (2018) [41], Stevens (2010) [44], Taylor (2021) [45]		
Adults utilizing coping strategies replicated from their own childhood	Lindberg (2024) [37]		
Adults distract themselves by avoiding interactions with others, sleeping, drinking, and spending more time with their children	Leung (2022) [34], Liebe (2024)	Adults utilizing distractions to cope with food insecurity	
First-come first serve during plating	Burke (2017) [27]	Adults managing food resources in the household	
Locked up or hid food to save it	Liebe (2022) [24], Lindberg (2024) [37]		
Avoided having guests to avoid serving food	Liebe (2022) [24]		
Setting rules around food for picky eaters and giving priority to older children	Leung (2022) [34], Lindberg (2024) [37]		
Adults reduce children’s portion sizes	Nalty (2013) [39]		
Parents implementing strategies to break up monotony (adding variety, sitting down together at mealtime, adding desserts or sweets to a meal)	Rosa (2018) [41]		
Use cereal in the milk to help with baby’s hunger	Stevens (2010) [44]	Strategies to prevent or reduce hunger	
Provide or consume more snacks to ease hunger	Burke (2017) [27]		
Working extra to earn enough income to support family	Borre (2010) [26], Frongillo (1997) [29], Liebe (2024), Rosa (2018) [41], Zekeri (2007) [47]	Financial coping strategies	Financial coping strategies
Budget finances to stretch funds over a month	Burke (2017) [27], Kinsey (2019) [33], Quandt (2004) [40]		
Use utility and other bill money for food purchases	Burke (2017) [27], Gross (2019) [31], Hoisington (2002) [32], Kinsey (2019) [33], Liebe (2022) [24], Liebe (2024)		
Sell plasma for money to buy food	Burke (2017) [27]		
Making a choice between food and other needed supplies or services	Hoisington (2002) [32]		
Getting cash advances	Hoisington (2002) [32]		
Cutting food budget to afford other expenses	Leung (2022) [34]		
Adults stealing food	Rosa (2018) [41]		
Sold food or pawned any personal property	Liebe (2022) [24]		
Cohabiting	Zekeri (2007) [47]	Relying on support from family members or friends	Relying on support from family members or friends
Doubling-up (coresiding with a friend or relative)	Zekeri (2007) [47]		
Eating at family or friend’s house	Smith (2008) [44], Tomayko (2017) [46]		
Social support from friends and families to watch children so they can work	Lindberg (2024) [37]		
Pooling resources with others	Gorman (2017) [30], Gross (2019) [31], Kinsey (2019) [33]		
Exchanging or trading resources with others	Gorman (2017) [30], Hoisington (2002) [32], Kinsey (2019) [33]		
Borrow food (e.g., from family, friends, neighbors)	Burke (2017) [27], Gorman (2017) [30], Gross (2019) [31], Hoisington (2002) [32], Kinsey (2019) [33], Leung (2022) [34], Liebe (2022) [24], Rosa (2018) [41], Sharkey (2013) [42]		
Borrow money (e.g., from family, friends, neighbors)	Burke (2017) [27], Frongillo (1997) [29], Gorman (2017) [30], Gross (2019) [31], Kinsey (2019) [33], Leung (2022) [34], Liebe (2022) [24], Quandt (2004) [40], Rosa (2018) [41], Stevens (2010) [44], Zekeri (2007) [47]		
Children or teens engaged in illegal activities to acquire food (stealing food and other necessities, selling drugs, and "selling themselves")	Burris (2020) [28], Fram (2011) [22]	Children initiating their own strategies	Children implementing strategies to help maintain food-security status for the household
Children or teens participate in adult strategies to stretch resources	Fram (2011) [22], Leung (2020) [35]		
Child or teen got a job to provide for themselves or help their families	Burris (2020) [28], Fram (2011) [22], Leung (2020) [35]		
Children or teens initiate their own strategies to stretch resources (not asking for foods at the grocery store and asking only for healthy foods rather than treats)	Fram (2011) [22], Leung (2020) [35], Smith (2008) [43]		
Child distracts from or using imagination to cope with food insecurity	Leung (2020) [35]	Children engage in mental or emotional strategies to cope with food insecurity	
Appreciating parents for providing food and resources	Leung (2020) [35]		
Child or teens relied on help from teachers	Burris (2020) [28]	Children rely on support from community	
Child or teen turns to their community (churches, neighbors, friends, etc.)	Burris (2020) [28], Fram (2011) [22]		

Abbreviations: SNAP, Supplemental Nutrition Assistance Program; WIC, Special Supplemental Nutrition Program for Women, Infants, and Children.

### Food acquisition strategies

Food acquisition strategies were a common theme, which included shopping or purchasing techniques, using food assistance programs, and obtaining food through alternative sources. Caregivers purchased low-cost foods [24,26,27,[30], [31], [32], [33], [34],^38^,^40^,^41^,^46^], made changes in the foods that they purchased or obtained [27,32,33,40], bought fewer fruits and vegetables [24,26,30,33,34,37,46], reduced the quantity or quality of meats purchased [27,32,37], and cut other foods they considered expensive from the household food budget [27,32]. Using coupons, buying generic or store brands, and taking advantage of sales or discount offers were common budgeting techniques used [26,27,30,32,33,36,44]. To get the best deals, caregivers visited multiple stores [30,32,33,46] and shopped at budget grocery stores such as Walmart, Aldi, and Save-a-Lot [27,30,40,36,42]. Buying foods in bulk, especially meats and other protein sources [27,30,32,33,36], and stocking up on food and other necessities when extra money was available [33,41], were used to maintain food access throughout the month. Making a shopping list [30,31,33] and planning meals each week prevented unnecessary purchases and reduced food waste [27,31,33,41,44]. Other less commonly mentioned shopping techniques included lowering food expenditures by not eating out [29,31,33], purchasing less junk food [30], and stretching food dollars [40]. Additional food acquisition strategies included participation in federal nutrition assistance programs such as the Supplemental Nutrition Assistance Program (SNAP), Special Supplemental Nutrition Program for Women, Infants, and Children, Head Start, the National School Lunch Program, or the School Breakfast Program [23,24,26,27,[29], [30], [31],^33^,^34^,[36], [37], [38], [39], [40],^42^,^44^,^47^]. Community assistance programs helped to maintain food-security status through the services provided by local churches or food banks for emergency foods (18 studies) [24,26,27,[29], [30], [31], [32], [33], [34],[36], [37], [38],^40^,^41^,^42^,^44^,^46^,^47^], eating at a senior meal program (2 studies) [30,47], and visiting a social or community event to eat [24]. Acquiring food by using alternative sources supplemented a household’s food supply, as reported in some studies. These actions included hunting, fishing, or acquiring game [29,40] and gardening [27,29], along with domestic food production activities such as canning, raising meat, and food gathering or foraging for food [32]. One study focused on migrant seasonal farm workers documented that workers would take food from the field or eat it while still in the field [26], and gleaning crops from the field was also mentioned [41]. Another study reported using roadkill as a food source when available [32].

### Food management strategies

Food management strategies were a frequent theme that included techniques implemented during meal preparation and food consumption that impacted personal intake. Households prioritized the use of shelf-stable or fast foods over fresh foods by using canned goods, boxed dinners, and dollar menu items [24,27,29,30,[32], [33], [34],^44^,^46^]. Meals were stretched by preparing foods such as stews, soups, casseroles, pasta, beans, or rice dishes [27,31,32,[38], [39], [40], [41]], making use of leftovers and freezing meals for later use [27,[31], [32], [33],^40^,^41^], and substituting or using more canned goods [27,32,33,44]. Meals were simplified [27], made smaller [24,47], and ingredients were used to the fullest extent by removing spoiled parts from fruits or vegetables so that the unspoiled parts could still be used [24]. Parents and caregivers used portioning and serving methods to stretch food, such as serving smaller portions during mealtime [24,27,38], adults reducing children’s portion sizes [39], serving one portion first and allowing children to get seconds [34,37], serving on a first-come, first-served basis [27], setting rules around food for picky eaters [34,37], and giving priority to older children [37]. Adults also reported that they locked up or hid food to save it [24,37] and did not have guests over to avoid serving food to those outside of the family [24]. Caregivers got creative by preparing new meals or using what was available [27,[31], [32], [33]], having breakfast foods at any time of day [27], prioritizing child food preferences [27,34], as well as changing meal patterns, times, and frequency [27]. Parents also added variety to break up monotony of frequently prepared foods, sat down together at mealtimes, and added desserts or sweets to a meal [41]. Adults attempted to prevent or reduce hunger by providing more snacks to children [27] and adding cereal to milk for more filling infant feedings [44].

Certain household members reported several strategies that they personally implemented, which impacted their individual intake. For parents or caregivers, these were adults reducing their own portions or not eating at all [27,28,30,33,[35], [36], [37], [38],^41^,^44^,^45^,^47^], eating as much as possible when food was available [24], eating something else if there was not enough of the meal to eat [45], and prioritizing children eating first [23,24,36,37,41,44,45]. To distract themselves from the situation of food insecurity, adults reported avoiding interactions with others, sleeping more, drinking alcohol, or spending more time with their children to divert focus from their hunger [34,36]. In addition, adults reported that they would use approaches from their own childhood to maintain food-security status [37]. Children aged 6 to 14 also participated in changes to their personal intake, including skipping meals [35,39], going a full day without eating [35,39] when there was not enough money to buy food, relying on cheap foods such as fast food, snacks, and candy [28,43], and overeating at mealtimes to avoid hunger later [43]. If hungry, children would eat anything [43] or foods that were not preferred [43].

### Financial coping strategies

Financial coping strategies aside from food purchasing were implemented by adults or caregivers to maintain food-security status. Six studies reported that adults used money reserved for utilities or other bills to purchase food [23,24,27,[31], [32], [33]], whereas 1 study found that households acquired money for bills by reducing their food budget [34]. Low-income households may be faced with a choice between food and other necessary supplies or services [32]. Careful budgeting of household finances stretched funds for the month for food purchasing [27,33,40]. To afford food, adults sometimes worked additional shifts or hours, and multiple adults in the household engaged in working [26,29,36,41,47]. Other ways of ensuring there was enough money to purchase food included selling plasma [27], getting cash advances [32], stealing food [41], and selling food or pawning personal property [24].

### Reliance on support from family members or friends

Reliance on family members or friends was another theme with 8 distinct coping strategies where low-income households borrowed money (11 studies) [24,[27], [29], [30],^31^,^33^,^34^,^40^,^41^,^44^,^47^] and food (9 studies) [24,27,[30], [31], [32], [33], [34],^41^,^42^] from family, friends, and neighbors, others reported the practice of pooling resources between households by sharing meals or combining food and other resources [30,31,33], as well as exchanging resources with others [30,32,33]. Eating at a family member or friend’s house appeared in 2 studies [43,46], and another study mentioned that social support from friends and families was important to have children supervised while they work [37]. Cohabiting [47] or doubling-up [47] by coresiding with a friend or relative was a livelihood strategy for food insecurity.

### Strategies implemented by children

A key theme observed in 5 studies among children in low-income households was that they engaged in their own techniques to help maintain the household’s food security. Children described not asking for foods at the grocery store or asking only for healthy foods rather than treats [22,35,43]. Children also participated in adult approaches such as using leftovers and saving food to stretch resources [22,35]. They worked at jobs [22,28,35] and engaged in illegal activities such as stealing and selling drugs to acquire food for themselves or their families [22,28]. Obtaining support from community was another way that children coped by asking for help from teachers [28] and turning to members of their community, such as churches, neighbors, and friends, for support [22,28]. Children exercised mental or emotional tactics to cope with food insecurity by using their imagination or distracting themselves with music and other activities [41].

### Reported effectiveness of coping strategies

Of the 25 included studies, 8 explored the participant-reported effectiveness of coping strategies in maintaining food-security status through cross-sectional surveys, as well as individual and focus group interviews [23,26,31,33,40,41,42,44]. Participants reported approaches such as the use of federal nutrition assistance programs [31,33] and supplementing food supply with wild game [40] as effective during semistructured interviews. One study using the 2009 Colonia Household and Community Food Resource Assessment survey reported that child hunger was significantly reduced in households that used SNAP and bought food from neighbors or friends [42]. Results of the 18-item USDA Household Food Security Module in 1 study found that the use of multiple behavioral food coping strategies [23], such as purchasing cheaper foods and asking family members for food or money, was effective in shielding children from food insecurity. Semistructured interviews were carried out to show that those implementing several tactics [44], such as preparing more satisfying meals with strategies that increase food availability, making food more desirable for children, and using meal preparation techniques [41], were considered effective from the participants’ standpoint. One study focused on migrant seasonal farmworkers conducted focus group and individual interviews and found that participants reported that their risk of food insecurity was less in the United States than in their home country due to the strategy of working extra hours when available [26].

## Discussion

This scoping review identified 82 coping strategies recognized in recent literature, grouped into 5 themes that low-income families with children in the United States reported using to maintain food security and explored how these patterns related to the experiences of certain family members and in some cases, whether individuals thought they were effective. The included studies largely focused on the strategies that mothers and fathers implement to manage resources, suggesting that parents are the household members who most often practice coping techniques. Parental strategies were implemented while shopping or procuring foods, preparing meals, and during mealtimes to maintain food security by prioritizing availability of food, preventing hunger, and ensuring access to nutritious foods. The hypothesis that parents use coping techniques to shield children from experiencing food insecurity was supported by consistent reporting of approaches that prioritize children eating first and considering child preferences during food purchasing and meal preparation. Children and adolescents also practiced strategies to maintain food security for themselves and other members of the household by implementing their own distinct techniques or replicating adult patterns. The use of coping strategies varied by age group, with younger children and adolescents adopting adult techniques such as skipping meals and using leftovers, whereas teens may help acquire food by getting a job or stealing.

Previous studies suggest that adults and children in food-insecure households experience greater nutrient inadequacies in comparison with their food-secure peers [[5], [6], [7], [8]]. This review identified several coping strategies implemented during shopping, such as relying on low-cost, shelf-stable foods [24,26,27,30,31,32,33,34,38,40,41,46], buying fewer fruits and vegetables [24,26,30,33,34,37,46], and reducing the quality of meats purchased [27,32,37], which may contribute to these nutrient inadequacies. These purchasing techniques suggest that individuals may forgo some more healthful foods and food types to prioritize price and in certain situations foods with higher sodium intake, less of the nutrients in fruits and vegetables, and potentially higher saturated fats may be selected. These shifts, when accumulating over time, may shape dietary preferences and habits among children [48] and could increase the risk of diet-related chronic diseases later in life [18]. In addition to nutrient inadequacies, food insecurity is also linked with poor dietary quality in adults yet potentially less consistently in children [5]. This observation could result from strategies in which adults prioritized children eating first [23,24,[36], [41],^37^,^44^,^45^] and would eat something else if there was not enough of the meal to eat [45], suggesting that nutritious foods are initially designated to children when available. Several studies also reported practices that might be associated with improved diets, such as purchasing less junk food [30], gardening [27,29], children asking only for healthy foods [22], and cooking at home rather than eating out [31,33]. These approaches indicate that food-insecure households with children may also implement health-promoting behaviors, making it erroneous to conclude that all coping strategies may result in poorer nutrition or dietary quality.

The strategies identified in this review were aimed at managing the situation of food insecurity. For example, some households relied on alternative sources for food through hunting, fishing, acquiring game [29,40], gardening [27,29], and gathering roadkill [32]. These activities may increase food availability, prevent hunger, and support access to fruits and vegetables [49]. However, some of these methods could be linked to food safety risks, increasing exposure to infectious diseases, chemicals, and toxic metals found in wild game and noncommercially caught fish [49]. Other techniques, such as stealing, presented potential safety or legal risks, whereas actions such as using community resources or nutrition assistance programs were potentially positive in supporting food access and building supportive social networks. A range of circumstances may limit the ability to implement coping strategies. For example, recently relocated families may lack social support, households in urban areas may have limited access to alternative sources of food such as gardening or hunting, and those without fully documented status may not have access to federal nutrition assistance programs. Several techniques were used to prevent or manage hunger by providing more snacks to children [27], adding cereal to milk to make infant feedings more filling [44], as well as children eating anything [43] or foods that were not preferred [43]. These techniques may be beneficial in managing the situation of hunger, but eating anything or snacking more often could have negative impacts on diet quality.

Although prior studies have explored coping strategies in food-insecure households, their populations of focus differed from those in this review. Three published reviews investigated patterns among migrant seasonal farmworkers [50], ethnic or racial groups in the United States [51], and Malaysian populations [52]. These reviews identified some similar approaches compared with the findings in this study, such as using food assistance programs, altering personal intake patterns, consuming low-cost foods, relying on friends and family members, and implementing financial strategies. One review also reported that supplementing the food supply by consuming wild game or fish [50] was positively associated with food security. The other 2 reviews recognized new strategies, such as drinking fluids when hungry [52] and relying on low-cost, carbohydrate-rich foods [51], which add to the findings here. To the author’s knowledge, this is the first review to include summarizing the techniques used by children in food-insecure households.

The results highlight opportunities for nutrition education interventions to build on coping strategies already used by food-insecure households with children, including food acquisition or food management approaches as well as reliance on family members or friends. Future interventions may promote positive coping strategies to improve food-security status and dietary quality by emphasizing the importance of the nutritional quality of foods obtained. The reported effectiveness of federal nutrition assistance programs suggests the need for interventions that support program enrollment, retention, and benefit optimization, while also informing policies that enhance benefit adequacy and access to nutritious foods. For example, a previous study reported that interventions offering information with reminders through postal mail regarding potential SNAP eligibility as well as phone application assistance with a trained individual were associated with increased SNAP enrollment and benefit utilization [53]. However, there is limited evidence on interventions aimed at promoting continued participation in SNAP through benefit renewal. Future studies could determine if providing assistance with renewal by phone, similar to studies that offered support during initial enrollment, can effectively help households maintain SNAP. Although SNAP participation has been linked with improved food security, additional healthy incentive programs have been implemented to improve access to nutritious foods [54]. A previous review found that SNAP fruit and vegetable incentive programs are linked with increased purchasing and consumption of fruits and vegetables among low-income households in the United States [55]. Together, future interventions may potentially operationalize these opportunities by offering assistance with SNAP enrollment and renewal of benefits, along with fruit and vegetable incentive programs, to improve food security and dietary quality among low-income households with children. Given the role of family members and social networks, community-based programs could be designed to complement informal support systems and strengthen local food resources and their relationships with potential clients. Because children may actively participate in coping strategies, age-appropriate nutrition education should be incorporated to encourage healthy behaviors without transferring responsibility for food security to children. Collectively, these findings underscore the importance of multilevel approaches that integrate household nutrition education, community support, and policy action to improve food security among households with children.

This review has several strengths and limitations. A strength is the compilation of coping strategies used by both adults and children in low-income or food-insecure households, adding a first-time summary of this information. These findings may be valuable for informing future nutrition education interventions by promoting positive approaches that adults in low-income households reported as effective in managing food insecurity, such as using nutrition assistance programs and implementing techniques during meal preparation and the strategies that might also improve dietary quality, such as preparing food at home and buying less junk foods. A majority of these studies evaluated food-security status using the 18-Question United States Household Food Security Survey Module (HFSSM), enhancing comparability of findings, whereas others implemented the 6-Question Short Form HFSSM or the Radimer/Cornell survey. However, only 2 of the 5 studies focused on children or teens used a food-security survey that was intended for this audience to complete without adult assistance, such as the USDA’s Self-Administered Food Security Survey Module for Children Ages 12 Years and Older. In addition, all included studies were observational, with a majority employing a cross-sectional design. Few studies reported on the effectiveness of coping strategies in maintaining food security, and those that did collected this information through qualitative interviews with adult participants, limiting the ability to establish causal relationships. Participants may be reluctant to report techniques that are illegal or considered socially unacceptable, limiting understanding of the full range of coping strategies that food-insecure households with children implement. Studies that explored coping strategies for food insecurity during the COVID-19 pandemic were excluded because the situation regarding access to food and food insecurity was very different during COVID-19 compared with nonpandemic situations, and this review focused on food insecurity as the main exposure. Therefore, although families may have used coping strategies during the pandemic, these techniques may not have reflected those only related to food insecurity, but also those related to avoiding the spread of infectious disease.

In conclusion, in food-insecure households with children, a range of family members implement coping strategies aimed at managing the situation of food insecurity by increasing food availability and access to healthy foods. These strategies include food acquisition, food management, financial coping, reliance on support from family members or friends, and strategies implemented by children. Adults in food-insecure households with children reported that some of these approaches were effective at maintaining or improving food security, such as the use of federal nutrition assistance programs, support from family members and friends, and alternative sources of food. The findings of this review provide insights for future nutrition education interventions to improve food security in households with children.

## Author contributions

The authors’ responsibilities were as follows – ORR-B, HAE-M: designed the research plan; BM, ORR-B, HAE-M: designed the search strategy; ORB, HAE-M, AM-J: screened the studies for inclusion; ORR-B: wrote the paper; HAE-M, BM; AM-J: critically reviewed the paper; HAE-M: primary responsible for final content; and all authors: read and approved the final manuscript.

## Data availability

Data described in the manuscript are available in Table 1.

## Declaration of Generative AI and AI-assisted Technology in the Writing Process

The authors declare that no generative AI or AI-assisted technologies were used in the writing of this manuscript.

## Funding

This study was supported by the USDA’s National Institute of Food and Agriculture, Agriculture and Food Research Initiative Competitive Grants Program, Foundational and Applied Science Program grant no. 2022-68015-36279/project accession no. 1027912, USDA Hatch Project (grant 2021-IND90005789), and the Danone International Prize for Alimentation from the Danone Institute International Association and the French Foundation for Medical Research to HAE-M as the 2023–2024 Laureate of the Danone International Prize for Alimentation. Any opinions, findings, conclusions, or recommendations expressed in this publication are those of the authors and do not necessarily reflect the view of these organizations.

## Conflict of interest

HAE-M is a member of the *Advances in Nutrition* editorial board and played no role in the Journal’s evaluation of the manuscript. The other authors report no conflicts of interest.
